# Gray Matter Alterations in Pediatric Schizophrenia and Obsessive-Compulsive Disorder: A Systematic Review and Meta-Analysis of Voxel-Based Morphometry Studies

**DOI:** 10.3389/fpsyt.2022.785547

**Published:** 2022-03-02

**Authors:** Jingran Liu, Fang Wen, Junjuan Yan, Liping Yu, Fang Wang, Duo Wang, Jishui Zhang, Chunmei Yan, Jiahui Chu, Yanlin Li, Ying Li, Yonghua Cui

**Affiliations:** Department of Psychiatry, Beijing Children's Hospital, Capital Medical University, National Centre for Children's Health, Beijing, China

**Keywords:** schizophrenia, obsessive-compulsive disorders, activation likelihood estimation, gray matter, children and adolescents

## Abstract

**Objective:**

The aim of this study is comparing gray matter alterations in SCZ pediatric patients with those suffering from obsessive-compulsive disorder (OCD) based on a systematic review and an activation likelihood estimation (ALE) meta-analysis.

**Methods:**

A systematic literature search was performed in PubMed, Elsevier, and China National Knowledge Infrastructure (CNKI). A systematic review and an ALE meta-analysis were performed to quantitatively examine brain gray matter alterations.

**Results:**

Children and adolescents with schizophrenia had decreased gray matter volume (GMV) mainly in the prefrontal cortex (PFC), temporal cortex (such as the middle temporal gyrus and transverse temporal gyrus), and insula, while children and adolescents with OCD mainly had increased GMV in the PFC and the striatum (including the lentiform nucleus and caudate nucleus), and decreased GMV in the parietal cortex.

**Conclusions:**

Our results suggest that gray matter abnormalities in the PFC may indicate homogeneity between the two diseases. In children and adolescents, structural alterations in schizophrenia mainly involve the fronto-temporal and cortico-insula circuits, whereas those in OCD mainly involve the prefrontal-parietal and the prefrontal-striatal circuits.

## Introduction

Schizophrenia (SCZ), a severe psychiatric disorder characterized by symptoms such as hallucinations, delusions, disorganized thinking, amotivation, and cognitive dysfunction, has an onset in childhood and adolescence (1). Another serious psychiatric disorder that also often onsets in childhood and adolescence is obsessive-compulsive disorder (OCD), which is characterized by intrusive thoughts and repetitive and ritualistic behaviors (2). High comorbidity of OCD has been reported among patients with schizophrenia (3). A prior diagnosis of OCD and age of <20 years at OCD onset are associated with higher rates of subsequently diagnosed schizophrenia (4, 5). Some studies have found that SCZ and OCD share some demographic and clinical characteristics (6). These findings suggest that SCZ and OCD share common neuropathology. Therefore, many studies have compared SCZ and OCD to investigate their multidimensional heterogeneity.

Both SCZ and OCD have been recognized as neurodevelopmental disorders (7). Brain structural and functional abnormalities have been observed in SCZ and OCD at the early stages of life (8, 9). Indeed, some neuroimaging studies have compared brain structural abnormalities in adults with SCZ and OCD; however, the results of these studies are largely inconsistent. For example, Zhang et al. found that patients with SCZ and those with OCD lost similar gray matter (GM) volume in the right anterior cingulate (10). However, another study suggests that compared with patients with OCD, those with SCZ had reduced GM volume mainly in the prefrontal gyrus (including the left precuneus, left superior frontal gyrus, right middle frontal gyrus, etc.) (11). The above studies indicate that further investigations are warranted to compare gray matter volume differences between SCZ and OCD.

Notably, a meta-analysis of imaging studies (i.e., activation likelihood estimation [ALE] analysis) might serve as an important tool to confirm the structural and functional abnormalities in SCZ and OCD. Previously, to investigate the differences between SCZ and OCD, Goodkind et al. (12) performed a voxel-based morphometry (VBM)-based meta-analysis of 193 studies. They reported GM loss in the dorsal anterior cingulate cortex (dACC) and bilateral insula, brain areas that relate to executive functions. A secondary analysis of mega- and meta-analytical findings revealed that regions such as the hippocampus and fusiform gyrus exhibited high conformity to the shared morphometric signature of SCZ and OCD (13). Nevertheless, since existing research is limited to comparisons of adult populations, the similarities and differences in GM alterations among children and adolescents with either SCZ or OCD remain largely unknown. Indeed, previous studies found widespread structural brain changes in both pediatric OCD and adult OCD, but different age stages might indicate different structural alterations (14, 15). For example, by assessing cortical thickness and surface area, Boedhoe et al. (16) found that the parietal cortex was consistently implicated in both adults and children with OCD, but the temporal and frontal cortex changes were different during different stages of development and illness.

It should be noted that few studies have compared brain structural abnormalities in children and adolescents with SCZ and those with OCD. To the best of our knowledge, only one comparative study reported that children and adolescents with SCZ have more widespread white matter abnormalities than those with OCD (17). Children and adolescents with SCZ generally have decreased cortical GM, particularly in the frontotemporal cortical areas (18, 19). On the other hand, GM alterations present not only in the classical fronto–striatal–thalamic circuit but also in the parietal and occipital cortices have been found in pediatric OCD (14, 16, 20). However, further meta-analytical research into GM alterations in children and adolescents with SCZ and those with OCD is required.

Currently, there is no comparison between the two patient groups (SCZ and OCD) is performed in children and adolescents. The aim of this study is comparing gray matter alterations in SCZ patients with those suffering from OCD. First, a systematic review was performed to summarize the gray matter alterations in both SCZ and OCD. Second, an ALE meta-analysis was performed to quantitatively examine brain gray matter alterations. We hypothesized that shared GM alterations between SCZ and OCD should be within GM loss in the fronto–striatal–thalamic circuit. Thus, we intend to provide some neural indicators for children and adolescents with SCZ and those with OCD.

## Materials and Methods

### Literature Search

Literature searches were performed in online databases, including PubMed, Web of Science and China National Knowledge Infrastructure (CNKI). We used the keywords and combinations of the following search terms with the following search expressions: (“schizophrenia” OR “obsessive-compulsive disorder”) AND “structural” AND “MRI” AND “gray matter.” Additionally, the reference lists of relevant articles were obtained and screened for any additional studies missed by the database search. Next, the titles and abstracts of the articles identified were screened according to the inclusion criteria. After this screening stage, the full journal articles were checked to determine whether they met the criteria of the included studies. Studies were independently cross-checked by two researchers to identify relevant articles. Articles published up to 31 August 2021 were included.

### Inclusion and Exclusion Criteria

The following inclusion criteria were used to identify relevant studies for our meta-analysis: (1) English or Chinese language studies from peer-reviewed journals, (2) age of patients at diagnosis of SCZ or OCD (age: <18 years), and (3) VBM studies (both the whole brain analyses and ROI analyses were included). The exclusion criteria were as follows: (1) patient sample size <10 and (2) duplicate studies.

### Systematic Review and ALE Meta-Analysis

First, a systematic review was performed. The key step of the systematic review is data extraction. The extracted data included the “Sample Size,” “Age,” “Males Percentage,” “Duration of Illness (DOI),” as well as the “Brain regions of Gray matter alterations” (if the gray matter decreased, Patients < Controls, it will be marked A; if Patients> Controls, B was marked).

Second, based on the inclusion and exclusion criteria for the included studies, an ALE analysis was performed. The last BrainMap application, the Java-based version of Ginger ALE, is used for performing activation likelihood estimation (ALE) meta-analyses on sets of coordinates extracted from the database in Talairach or MNI space. In the present study, Ginger ALE version 3.0.2 was adopted in the present meta-analysis of the included VBM studies that reported the foci of GM changes (21). ALE analyses were conducted in Montreal Neurological Institute (MNI) space; however, if coordinates were originally reported in Talairach space, they were converted into MNI space with the Lancaster transform using the icbm2tal transformation function implemented within GingerALE (22). The resulting statistical maps were corrected for a threshold at *p* < 0.001 (False Discovery Rate correction, FDR) with a cluster extent threshold of 50 voxels. For visualization, whole-brain maps of threshold ALE maps were imported into multi-image analysis Mango (http://ric.uthscsa.edu/mango) and overlaid onto a standardized anatomical template (the ICBM-152 brain template) (22).

## Results

### Identification of Included Studies

**Figure 1:**
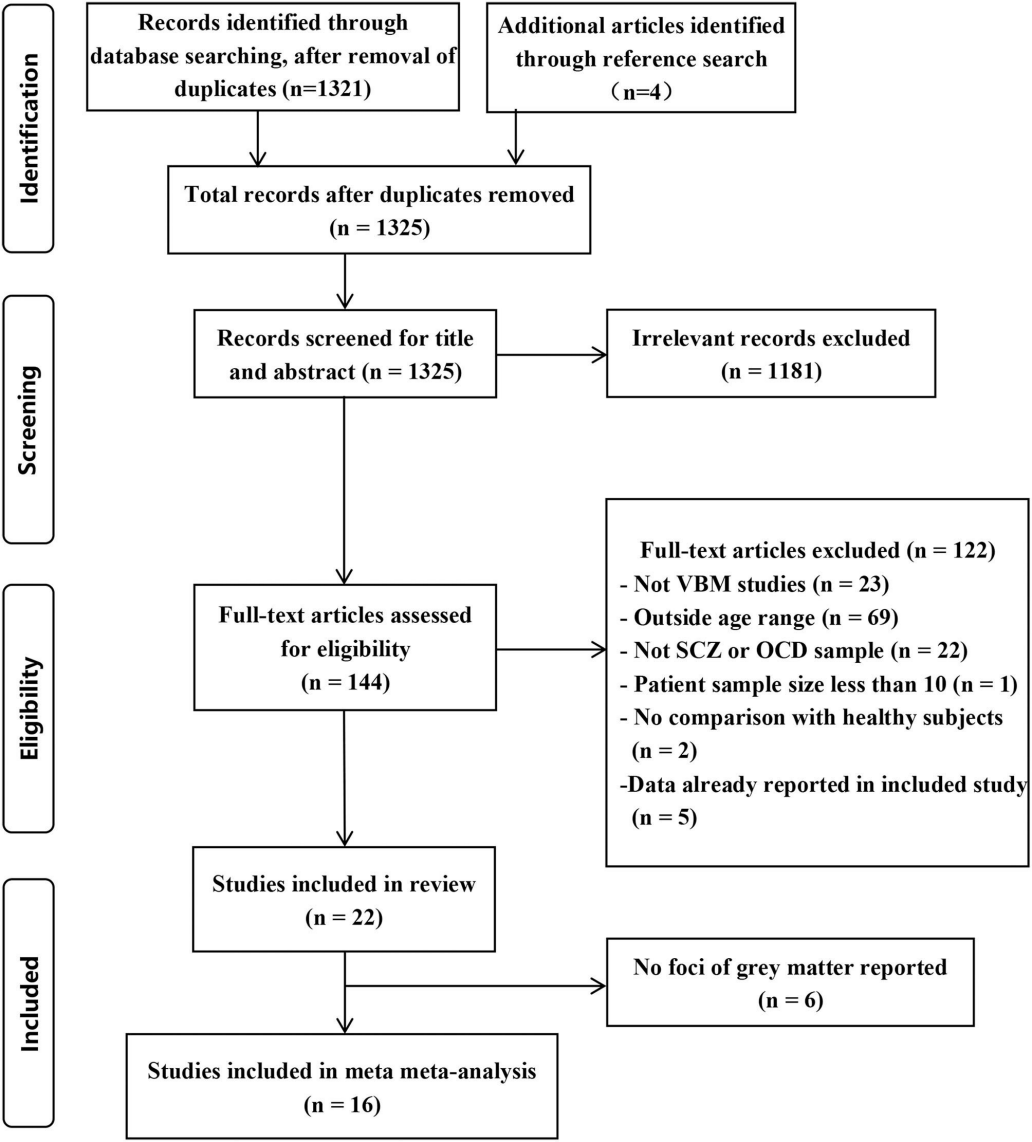
Flowchart of the selection criteria.

The identification procedure of the studies can be found in Figure 1. Initially, we identified 21 English language studies and 1 Chinese language study according to the criteria. We list the exclusion information in Supplementary Table 1.

### Systematic Review of Included Studies

The VBM datasets were obtained from 11 SCZ studies and 11 OCD studies. The baseline characteristics of all participants and the brain regions of gray matter alterations are summarized in Table 1. Two SCZ studies found GM volume changes without reporting the foci (23, 29). Four studies did not find any significant differences in GM volume between patients and healthy controls (26, 33, 36, 38). It seems that children and adolescents with schizophrenia had decreased gray matter volume (GMV), mainly in the prefrontal cortex (PFC), temporal cortex and insula, while children and adolescents with OCD mainly had increased GMV in the PFC and the striatum, but decreased GMV in the parietal cortex.

**Table 1:** Demographic and clinical characteristics of the 22 voxel-based morphometry studies.

**References**	**Patients**					**Controls**			**Brain regions of gray matter alterations**	
	**Sample size**	**Age**	**Males%**	**DOI**	**Treatment**	**Sample size**	**Age**	**Males%**	**Patients < Controls (A)**	**Patients> Controls (B)**
SCZ studies										
Wen et al. (23)	29	14.93 ± 1.60	34	NR	NR	28	16.00 ± 0.47	43	Hippocampus (A)	
Gao et al. (24)	39	13.5 ± 2.3	44	5 ± 3	0	39	13.3 ± 2.0	36	R insula, L IFG, L limbic edge (A)	
Zhang et al. (25)	26	16.87 ± 1.05	50	3.61 ± 3.50	0	26	16.81 ± 0.75	50	L parietal postcentral gyrus, L parahippocampa (A)	
Castro-Fornieles et al. (26)	34	15.2 ± 1.7	71	NR	NR	70	15.3 ± 1.5	60	No positive results	
Zhang et al. (27)	37	15.5 ± 1.8	46	16.0 ± 14.4	0	37	15.3 ± 1.6	46	R STG, R MTG (A)	
Tang et al. (28)	29	16.5 ± 0.9	45	9.3 ± 4.6	79	29	16.6 ± 0.8	55	L STG, L MTG (A)	
James et al. (29)	32	16.3 ± 1.2	69	21.6 ± NR	100	28	16.4 ± 1.4	64	PFC, STG, ITG (A)	
Yoshihara et al. (30)	18	15.8 ± 1.3	50	14.4 ± 10.8	94	18	15.8 ± 1.8	50	L parahippocampal, IFG, STG (A)	
Janssen et al. (31)	25	15.4 ± 1.8	76	3.5 ± 2.2	NR	25	15.4 ± 1.6	69	L medial frontal gyrus, L MFG (A)	
Douaud et al. (32)	25	16.3 ± 1.3	72	16.8 ± 8.4	100	25	16.0 ± 1.7	68	SMA, R ACC, R dorso-lateral PFC (A)	
Pagsberg et al. (33)	15	15.6 ± 1.8	47	NR	NR	29	16 ± 1.9	38	No Positive Results	
OCD studies										
Cheng et al. (34)	30	10.8 ± 2.1	60	NR	0	30	10.5 ± 2.2	60	L IPL(A), Putamen, L OFC (B)	
Jayarajan et al. (35)	15	14.13 ± 1.79	53	16.8 ± 12.9	86	15	14.31 ± 2.12	53	L ACC (A)	
Lázaro et al. (36)	62	15.4 ± 2.1	58	28.29 ± 24.16	84	46	15.3 ± 2.1	48	No Positive Results	
Huyser et al. (37)	29	13.78 ± 2.58	28	31.2 ± 27.6	0	29	13.60 ± 2.73	28	L superior frontal pole, L insula (B)	
Lázaro et al. (38)	27	15.6 ± 1.5	56	NR	100	27	16.1 ± 1.3	48	No Positive Results	
Zarei et al. (39)	26	16.6 ± 1.5	54	63.6 ± 40.8	62	26	16.5 ± 1.4	54	Caudate, R putamen (B)	
Britton et al. (40)	15	13.5 ± 2.4	60	49.2 ± 24.0	100	20	13.6 ± 2.4	65	Medial frontal gyrus, OFC; R ACC (B)	
Lázaro et al. (41)	15	13.7 ± 2.5	53	21.2 ± 16.6	0	15	14.3 ± 2.5	53	Parietal lobes(A)	
Szeszko et al. (42)	37	13.0 ± 2.7	38	43.2 ± NR	0	26	13.0 ± 2.6	35	Occipital cortex (A); OFC, STG, parietal lobe (B)	
Gilbert et al. (43)	10	12.9 ± 2.7	60	NR	0	10	13.4 ± 2.6	60	L ACC, medial SFG (A)	
Carmona et al. (44)	18	12.86 ± 2.76	72	NR	56	18	13.03 ± 3.04	72	Frontal lobe, cingulate cortex (A)	

*SCZ, schizophrenia; OCD, obsessive-compulsive disorder; y, years; m, months; DOI, duration of illness; SD, standard deviation; NR, not recorded; R, right; L, left; IFG, inferior frontal gyrus; STG, superior temporal gyrus; MTG, middle temporal gyrus; PFC, prefrontal cortex; ITG, inferior temporal gyrus; MFG, middle frontal gyrus; SFG, superior frontal gyrus; SMA, supplementary motor area; IPL, inferior parietal lobule; OFC, orbitofrontal cortex; ACC, anterior cingulate cortex*.

### ALE Analysis in Children and Adolescents With SCZ and OCD

**Figure 2:**
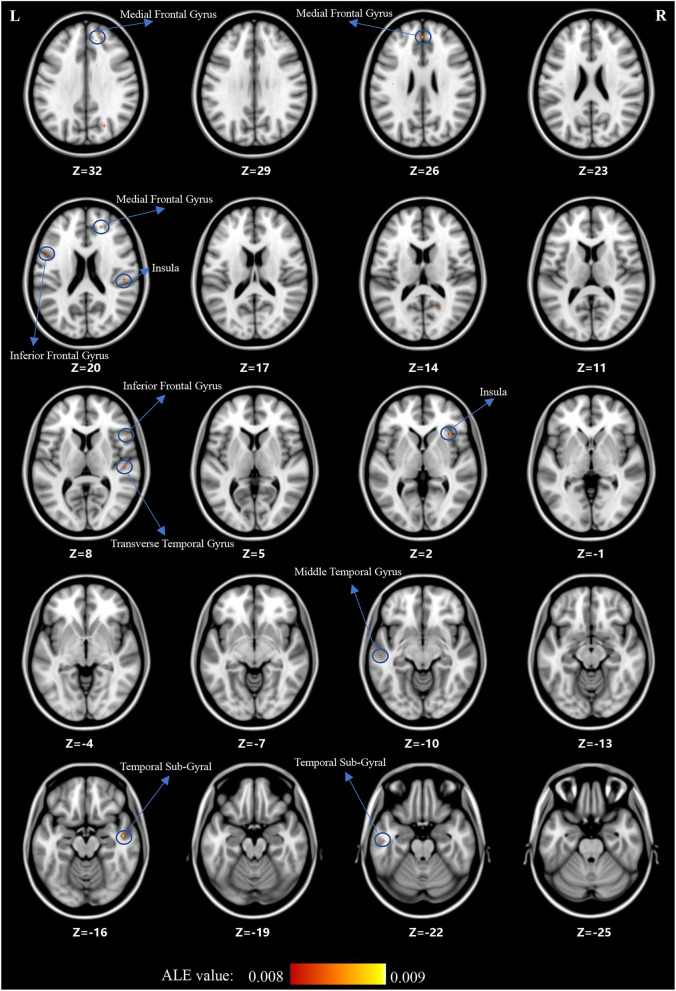
The gray matter reduction (red) in SCZ based on ALE analysis.

For the ALE analysis, there were 7 SCZ studies (including 199 patients with SCZ and 225 control subjects) and nine OCD studies (including 195 patients with OCD and 189 control subjects). ALE analysis in children and adolescents with SCZ revealed that GM volume was significantly reduced in the bilateral medial frontal gyrus, right middle frontal gyrus (MFG), bilateral inferior frontal gyrus (IFG), bilateral superior frontal gyrus (SFG), bilateral temporal sub-gyrus, and so on (for more details, see Table 2; Figure 2).

**Table 2:** Results of ALE analyses on gray matter reduction in SCZ.

**Cluster #**	**Volume (mm^**3**^)**	**Peak ALE value**	**MNI coordinates (x,y,z)**			**Brain regions**
1	96	0.009153046	48	−10	−16	(R) Temporal Sub-Gyral (BA21)
2	96	0.008924574	36	21	3	(R) Insula (BA13)
3	96	0.008930734	0	44	28	(L) Medial Frontal Gyrus (BA9)
4	80	0.009246408	54	18	9	(R) Inferior Frontal Gyrus (BA44)
5	64	0.008868549	−54	−22	−12	(L) Middle Temporal Gyrus (BA21)
6	56	0.008880154	−50	−16	−22	(L) Temporal Sub-Gyral (BA20)
7	56	0.008856174	−12	−90	6	(L) Lingual Gyrus (BA17)
8	56	0.008856174	50	−22	8	(R) Transverse Temporal Gyrus (BA41)
9	56	0.008856174	−48	−18	10	(L) Transverse Temporal Gyrus (BA41)
10	56	0.008856174	−46	18	12	(L) Inferior Frontal Gyrus (BA44)
11	56	0.008856174	20	−60	14	(R) Posterior Cingulate (BA30)
12	56	0.008856174	50	−24	20	(R) Insula (BA13)
13	56	0.008856174	−50	8	20	(L) Inferior Frontal Gyrus (BA9)
14	56	0.008856175	20	44	20	(R) Medial Frontal Gyrus (BA9)
15	56	0.008856174	−38	−16	24	(L) Insula (BA13)
16	56	0.008858921	14	40	30	(R) Medial Frontal Gyrus (BA9)
17	56	0.008856174	24	−70	32	(R) Precuneus (BA31)
18	56	0.008856176	20	44	32	(R) Superior Frontal Gyrus (BA9)
19	56	0.008856174	14	8	34	(R) Cingulate (BA24)
20	56	0.008856174	38	10	38	(R) Medial Frontal Gyrus (BA6)
21	56	0.008856174	60	−16	44	(R) Postcentral Gyrus (BA3)
22	56	0.008856174	−40	−36	46	(L) Inferior Parietal Lobule (BA40)
23	56	0.008856235	−22	18	48	(L) Superior Frontal Gyrus (BA6)
24	56	0.008929286	−10	−38	70	(L) Postcentral Gyrus (BA5)

*SCZ, schizophrenia; ALE, activation likelihood estimation; MNI, Montreal Neurological Institute; R, right; L, left; BA, Brodmann area*.

**Figure 3:**
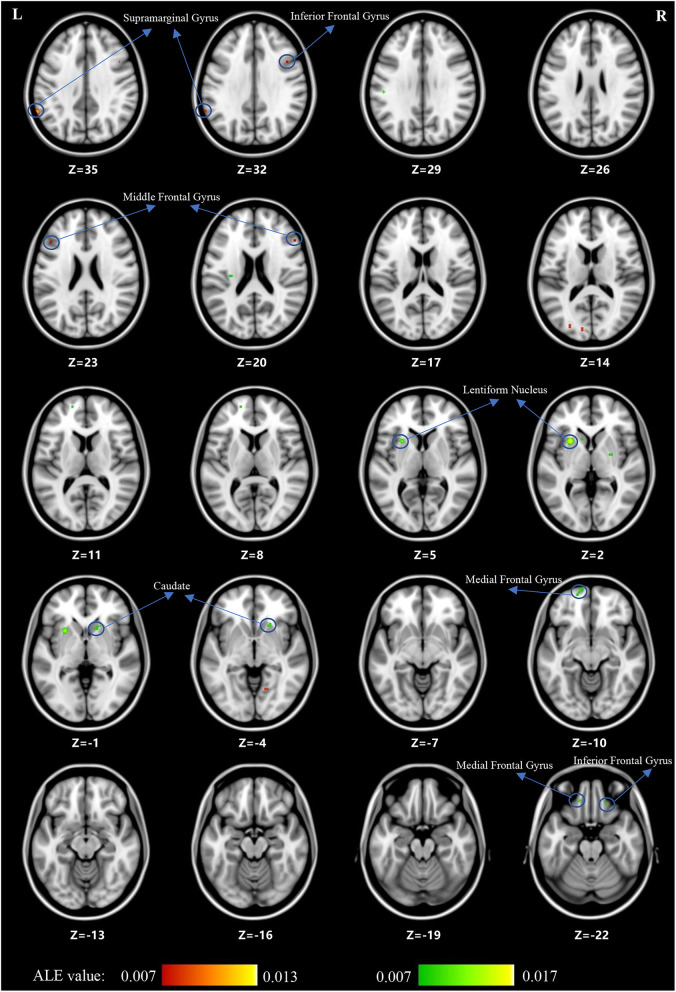
The gray matter reduction (red) and increase (green) in OCD based on ALE analysis.

For children and adolescents with OCD, GM volume was significantly reduced in the left supramarginal gyrus, left cuneus, left middle occipital gyrus, right IFG, right SFG, bilateral MFG, right precentral gyrus, right paracentral lobule, right precuneus, bilateral cingulate, and right culmen. Simultaneously, GM volume was significantly increased in the left medial frontal gyrus, right MFG, right IFG, left SFG, striatum and so on (for more details, see Tables 3, 4; Figure 3).

**Table 3:** Results of ALE analyses on gray matter reduction in OCD.

**Cluster #**	**Volume (mm^**3**^)**	**Peak ALE value**	**MNI coordinates (x,y,z)**			**Brain regions**
1	312	0.013117323	−62	−52	38	(L) Supramarginal Gyrus (BA40)
2	96	0.008858794	−11	−88	13	(L) Cuneus (BA17)
3	96	0.008858794	−27	−84	13	(L) Middle Occipital Gyrus (BA18)
4	96	0.00766531	44	13	35	(R) Precentral Gyrus (BA9)
4		0.007424954	43	13	30	(R) Inferior Frontal Gyrus (BA9)
5	96	0.00766531	25	29	42	(R) Superior Frontal Gyrus (BA8)
5		0.007424954	27	24	43	(R) Superior Frontal Gyrus (BA8)
6	96	0.00766531	3	−13	48	(R) Paracentral Lobule (BA31)
6		0.007424954	8	−11	49	(R) Paracentral Lobule (BA31)
7	80	0.009176578	16	−63	−4	(R) Culmen
8	64	0.007424954	53	27	21	(R) Middle Frontal Gyrus (BA46)
9	64	0.007424954	−45	25	23	(L) Middle Frontal Gyrus (BA46)
10	64	0.007424954	15	−53	41	(R) Precuneus (BA31)
11	64	0.007424954	15	−37	43	(R) Cingulate Gyrus (BA31)
12	64	0.007424954	1	−31	47	(L) Cingulate Gyrus (BA31)

*OCD, obsessive-compulsive disorder; ALE, activation likelihood estimation; MNI, Montreal Neurological Institute; R, right; L, left; BA, Brodmann area*.

**Table 4:** Results of ALE analyses on gray matter increase in OCD.

**Cluster #**	**Volume (mm^**3**^)**	**Peak ALE value**	**MNI coordinates (x,y,z)**			**Brain regions**
1	608	0.016521817	−26	12	2	(L) Lentiform Nucleus (Putamen)
2	304	0.009560066	20	20	−2	(R) Caudate (Caudate Head)
		0.008989162	14	16	−2	(R) Caudate (Caudate Head)
3	224	0.009225059	−12	66	−10	(L) Medial Frontal Gyrus (BA10)
		0.008012949	−18	58	−10	(L) Superior Frontal Gyrus (BA10)
4	200	0.00917098	−18	56	10	(L) Superior Frontal Gyrus (BA10)
		0.008015263	−8	56	10	(L) Medial Frontal Gyrus (BA10)
5	112	0.009076225	−14	38	−22	(L) Medial Frontal Gyrus (BA11)
6	96	0.008518396	−30	−19	19	(L) Claustrum
7	96	0.008858794	13	−52	63	(R) Precuneus Gray (BA7)
8	80	0.009176578	20	34	−23	(R) Inferior Frontal Gyrus (BA47)
9	80	0.008632486	26	−5	2	(R) Lentiform Nucleus (Putamen)
10	80	0.00881557	−50	−26	29	(L) Inferior Parietal Lobule (BA40)
11	80	0.009176578	8	−69	58	(R) Superior Parietal Lobule (BA7)
12	80	0.009176578	−12	−65	62	(L) Superior Parietal Lobule (BA7)
13	64	0.008552016	45	3	−25	(R) Superior Temporal Gyrus (BA38)
14	64	0.008079925	−12	14	2	(L) Caudate (Caudate Head)
15	64	0.008495986	32	3	45	(R) Middle Frontal Gyrus (BA6)

*OCD, obsessive-compulsive disorder; ALE, activation likelihood estimation; MNI, Montreal Neurological Institute; R, right; L, left; BA, Brodmann area*.

## Discussion

The current systematic review and ALE meta-analysis revealed that children and adolescents with either SCZ or OCD have significant GMV abnormalities in multiple brain regions. However, despite being relatively consistent with the existing literature, our findings showed heterogeneous results. First, both children and adolescents with SCZ and those with OCD showed GMV alterations in the prefrontal cortex (PFC), which included the medial frontal gyrus and MFG (BA9, BA10). Notably, in this area, GMV was decreased in children and adolescents with SCZ and increased in those with OCD. Second, children and adolescents with SCZ showed decreased GMV in the temporal cortex (especially in the MTG and the transverse temporal gyrus) and insula. However, for children and adolescents with OCD, loss of GM was found in the parietal cortex, mainly in the supramarginal gyrus. Third, children and adolescents with OCD had a greater striatal GM volume, including the lentiform nucleus and caudate nucleus, than control subjects. Overall, we found that children and adolescents with SCZ and those with OCD have significant GMV abnormalities in multiple brain regions. For the cortical cortex, a decrease in GMV was observed mainly in the areas of the PFC (medial frontal gyrus and MFG), temporal cortex (especially in the MTG and transverse temporal gyrus), and insula in children and adolescents with SCZ. In children and adolescents with OCD, an increase in GMV was observed in the PFC, while a decrease in GMV was observed in the parietal cortex (supramarginal gyrus). For subcortical regions, we found that children and adolescents with OCD had a greater striatal volume, including the lentiform nucleus and caudate nucleus.

Gray matter loss in the PFC has been previously reported in children and adolescents with SCZ. In addition to the VBM studies in our meta-analysis, studies using the “region of interest” (ROI) approach suggested that children and adolescents with SCZ had a deficit in GM volume in the PFC (45–47). In addition, longitudinal magnetic resonance imaging studies have reported that children and adolescents with SCZ showed greater progressive frontal GM loss over years after illness onset than healthy individuals (48, 49). Recently, a large-scale study concluded that individuals with schizophrenia have a widespread thinner cortex and smaller surface area in frontal lobe regions (50). Supplementing previous research, the results of this study indicate that GM loss in the PFC might occur at an earlier course of SCZ.

We found an increase, rather than a decrease, in GMV of the PFC in children and adolescents with OCD, in contrast to that reported in a previous meta-analysis (20). These discrepancies can be explained by the differences in the OCD studies included. We excluded a study reporting a lower PFC volume because of the small sample size (51) and two studies reporting a greater PFC volume (34, 40). Recently, a large-scale graph analysis of brain structural covariance networks found that the PFC exhibited OCD-related alterations in the trajectories of brain development and maturation (52). Similar to SCZ, the results of the present study also indicated that GM alterations might occur at an earlier course of OCD.

The PFC plays an essential role in the organization and control of goal-directed thoughts and behaviors (53). Furthermore, the PFC orchestrates a wide range of cognitive and affective neuronal functions thanks to its extensive reciprocal connections to nearly all cortical and subcortical structures (53). The PFC dysconnectivity pattern in patients with SCZ is associated with the severity of cognitive impairments (such as impaired working memory) (54). Disruption of executive functions that are PFC-regulated may lead to the generation of obsessions and compulsions in patients with OCD (55). Several functional imaging studies have consistently highlighted abnormal activity patterns in PFC regions and connected circuits in SCZ and OCD during both symptom provocation and performance of neurocognitive tasks (54, 55). Our results hint at GM alterations in the PFC shared by children and adolescents with SCZ and those with OCD, which might account for comorbid cognitive deficits in both disorders.

In terms of other cortical abnormalities, GMV in the temporal cortex was decreased in children and adolescents with SCZ, whereas GMV in the parietal cortex was decreased in children and adolescents with OCD. Previous studies have reported a decreased GMV in the temporal cortex as well as in the PFC (47, 48). Regions in the temporal lobe are associated with auditory hallucinations, thought disorder, and memory dysfunction and are key characteristics of schizophrenia (28). In a previous study, loss of GM in the temporal cortex was negatively correlated with positive symptoms in SCZ (28). Several studies have verified the relationship between frontotemporal functional dysconnectivity and auditory hallucinations during different tasks, suggesting a source-monitoring impairment (56). The parietal cortex has been continuously implicated in the pathophysiology of both adult and pediatric OCD (16, 57). It has been hypothesized that the repetitive behaviors in OCD reflect the problems in set-shifting (58), in which the supramarginal gyrus plays a key role (59). Moreover, the supramarginal gyrus is part of the inferior parietal lobule (IPL). As an important node in both the fronto-parietal network and the default mode network, the IPL is considered to underlie OCD symptoms, such as the inability to eliminate persistent intrusive thoughts (60). In general, GM deficits in the temporal cortex in SCZ patients are associated with positive symptoms, whereas GM deficits in the parietal cortex in OCD patients may be the basis of compulsive behavior. These results tap into the heterogeneity of the two diseases.

Another key observation in this meta-analysis is that GM volume is reduced in the insula among children and adolescents with SCZ. A decrease in GM volume in the insula was found in adults with early-onset schizophrenia (61). A meta-analysis of ROI studies reported medium-sized bilateral GM volume reduction in the insular cortex in schizophrenia, which showed no progression with illness stage (62). Volume reduction in the insular cortex may constitute an important neuropathology in schizophrenia. Significant and widespread dysconnectivity of insula subregions is observed in schizophrenia, which correlates with cognitive function (63). Individuals with schizophrenia have impaired anterior insula-related large-scale brain networks, especially the central executive and default mode networks (64). The disrupted processing in the insula or a network involving the region could contribute to many sensory deficits found in schizophrenia. Failure of this process may lead to internally generated sensory information being attributed to an external source, which in turn contributes to hallucinations (65).

For subcortical regions, we found that the striatal volume was greater in pediatric OCD, consistent with findings of a previous meta-analysis (20). The aforementioned GM alterations in the PFC combined with our findings support theories of prefrontal–striatal circuit abnormalities in pediatric OCD (66). The prefrontal–striatal circuit, the main part of inhibitory control networks, includes several brain regions, such as the ventrolateral prefrontal cortex, anterior insula, supplementary motor area, dACC, and the striatal, thalamic, and dorsolateral prefrontal cortex (67). In OCD, deficits in inhibitory control were thought to underlie the poor control over obsessions and compulsions (68, 69). The prefrontal–striatal circuit is also part of the cortico-striatal-thalamo-cortical (CSTC) pathway. Hyperactivity in the CSTC pathway is thought to underlie the manifestation of OCD (70). Moreover, a meta-analysis of executive function in OCD showed that OCD is associated with broad impairments in executive function (71). Indeed, the impaired executive function in OCD also showed an association with the prefrontal–striatal circuit (55). Meanwhile, GMV in the striatum was greater in OCD than in attention-deficit hyperactivity disorder and autism spectrum disorder (72, 73). In addition, no alterations in striatal volume were found in children and adolescents with SCZ. The results suggest that greater striatal volume may be a disorder-specific neural structural biomarker of pediatric OCD relative to other psychiatric disorders.

Several limitations were noted in the current study. First, the number of studies in our ALE meta-analysis was small. Based on a recent simulation study (74), a recommendation was made to include at least 17–20 experiments in ALE meta-analyses to have sufficient power. However, the present schizophrenia meta-analysis is based on only seven studies, and the meta-analysis on OCD patients contains only nine studies. Therefore, the power can be assumed to be very weak, and the results can only be regarded as indicators for future studies. Second, Müller et al. (75) reported 10 simple rules for neuroimaging meta-analysis. One of the rules is that a cluster-level Family Wise Error (FWE) correction is recommended for ALE meta-analyses, but in the present study, a loose correction (FRD correction, *p* < 0.001) was used to obtain more results, which made our results preliminary and highly heterogeneous. Third, in line with most voxelwise meta-analyses, our study was based on brain coordinates extracted from published studies rather than raw statistical brain maps. This may also lead to less accurate results (76). Fourth, due to the limited sample size, we did not consider the influencing factors (e.g., treatment, age, and duration of illness). For example, it was consistently reported that both anatomical and functional brain components, including the frontal and temporal lobes, basal ganglia, limbic system and several key components within the default mode network, changed in patients with SCZ after antipsychotic treatment (77). The influence of antidepressants was also reported in OCD (78). However, the influence of medicines on the brains of patients with mental disorders might be an important direction for future studies.

## Conclusions

In children and adolescents with SCZ, GM alterations are observed in the PFC, the temporal cortex, and the insula. In children and adolescents with OCD, GM alterations are exhibited in the PFC and striatum. These results suggest that GM abnormalities in the PFC may be a good indicator of the homogeneity between these two disorders. It is suggested that the majority of children and adolescents with SCZ have core defects in the prefrontal-temporal and cortico-insula circuits, whereas those with OCD have core defects in the prefrontal-parietal and the prefrontal-striatal circuits.

## Data Availability Statement

The original contributions presented in the study are included in the article/Supplementary Material, further inquiries can be directed to the corresponding authors.

## Author Contributions

YC and YiL took the initiative. FWe, JY, LY, FWa, DW, JZ, CY, JC, and YaL finished the study search and data extraction. YiL performed the data analysis. JL finished the draft. All authors contributed to the article and approved the submitted version.

## Funding

This work was supported by the National Natural Science Foundation of China (NSFC) under (Grant Nos. 82001445 and 82171538) and the Beijing Natural Science Foundation under (Grant No. 7212035).

## Conflict of Interest

The authors declare that the research was conducted in the absence of any commercial or financial relationships that could be construed as a potential conflict of interest.

## Publisher's Note

All claims expressed in this article are solely those of the authors and do not necessarily represent those of their affiliated organizations, or those of the publisher, the editors and the reviewers. Any product that may be evaluated in this article, or claim that may be made by its manufacturer, is not guaranteed or endorsed by the publisher.
