# Supplementary material for: Gray Matter Alterations in Pediatric Schizophrenia and Obsessive-Compulsive Disorder: A Systematic Review and Meta-Analysis of Voxel-Based Morphometry Studies
Source: Front Psychiatry. 2022 Mar 2;13:785547. doi: 10.3389/fpsyt.2022.785547 (PMC8924120; doi:10.3389/fpsyt.2022.785547)
Supplement: Supplementary file 1 [file Data_Sheet_1.doc]

**Table S1. The Full-text articles excluded （total n =122）**

| Studies | n | Reason for exclusion |
| --- | --- | --- |
| [1-23] | 23 | Not VBM studies |
| [24-92] | 69 | Outside age range |
| [93-114] | 22 | Not SCZ or OCD sample |
| [115] | 1 | Patient sample size less than 10 |
| [116,117] | 2 | No comparison with healthy subjects |
| [118-122] | 5 | Data already reported in included study |

[1] Jiang Y, Luo C, Li X, et al. Progressive Reduction in Gray Matter in Patients with Schizophrenia Assessed with MR Imaging by Using Causal Network Analysis. Radiology, 2018, 287(2): 633-642.

[2] Cauda F, Nani A, Costa T, et al. The morphometric co-atrophy networking of schizophrenia, autistic and obsessive spectrum disorders. Hum Brain Mapp, 2018, 39(5): 1898-1928.

[3] Zhou HY, Shi LJ, Shen YM, et al. Altered topographical organization of grey matter structural network in early-onset schizophrenia. Psychiatry Res Neuroimaging, 2021, 316: 111344.

[4] Vattimo EFQ, Dos Santos AC, Hoexter MQ, et al. Higher volumes of hippocampal subfields in pediatric obsessive-compulsive disorder. Psychiatry Res Neuroimaging, 2021, 307: 111200.

[5] Shi LJ, Zhou HY, Wang Y, et al. Altered empathy-related resting-state functional connectivity in adolescents with early-onset schizophrenia and autism spectrum disorders. Asian J Psychiatr, 2020, 53: 102167.

[6] Vattimo EFQ, Barros VB, Requena G, et al. Caudate volume differences among treatment responders, non-responders and controls in children with obsessive-compulsive disorder. Eur Child Adolesc Psychiatry, 2019, 28(12): 1607-1617.

[7] Luo Q, Chen Q, Wang W, et al. Association of a Schizophrenia-Risk Nonsynonymous Variant With Putamen Volume in Adolescents: A Voxelwise and Genome-Wide Association Study. JAMA Psychiatry, 2019, 76(4): 435-445.

[8] Rich AM, Cho YT, Tang Y, et al. Amygdala volume is reduced in early course schizophrenia. Psychiatry Res Neuroimaging, 2016, 250: 50-60.

[9] Pina-Camacho L, Del Rey-Mejías Á, Janssen J, et al. Age at First Episode Modulates Diagnosis-Related Structural Brain Abnormalities in Psychosis. Schizophr Bull, 2016, 42(2): 344-357.

[10] Günther V, Lindner C, Dannlowski U, et al. Amygdalar Gray Matter Volume and Social Relating in Schizophrenia. Neuropsychobiology, 2016, 74(3): 139-143.

[11] Epstein KA, Kumra S. Altered cortical maturation in adolescent cannabis users with and without schizophrenia. Schizophr Res, 2015, 162(1-3): 143-152.

[12] Liu Y, Hanna GL, Carrasco M, et al. Altered relationship between electrophysiological response to errors and gray matter volumes in an extended network for error-processing in pediatric obsessive-compulsive disorder. Hum Brain Mapp, 2014, 35(4): 1143-1153.

[13] Sprooten E, Papmeyer M, Smyth AM, et al. Cortical thickness in first-episode schizophrenia patients and individuals at high familial risk: a cross-sectional comparison. Schizophr Res, 2013, 151(1-3): 259-264.

[14] Kumra S, Robinson P, Tambyraja R, et al. Parietal lobe volume deficits in adolescents with schizophrenia and adolescents with cannabis use disorders. J Am Acad Child Adolesc Psychiatry, 2012, 51(2): 171-180.

[15] Juuhl-Langseth M, Rimol LM, Rasmussen IA, Jr., et al. Comprehensive segmentation of subcortical brain volumes in early onset schizophrenia reveals limited structural abnormalities. Psychiatry Res, 2012, 203(1): 14-23.

[16] Reig S, Parellada M, Castro-Fornieles J, et al. Multicenter study of brain volume abnormalities in children and adolescent-onset psychosis. Schizophr Bull, 2011, 37(6): 1270-1280.

[17] Raznahan A, Greenstein D, Lee Y, et al. Catechol-o-methyl transferase (COMT) val158met polymorphism and adolescent cortical development in patients with childhood-onset schizophrenia, their non-psychotic siblings, and healthy controls. Neuroimage, 2011, 57(4): 1517-1523.

[18] Parellada M, Boada L, Fraguas D, et al. Trait and state attributes of insight in first episodes of early-onset schizophrenia and other psychoses: a 2-year longitudinal study. Schizophr Bull, 2011, 37(1): 38-51.

[19] Greenstein D, Lerch J, Shaw P, et al. Childhood onset schizophrenia: cortical brain abnormalities as young adults. J Child Psychol Psychiatry, 2006, 47(10): 1003-1012.

[20] Moreno D, Burdalo M, Reig S, et al. Structural neuroimaging in adolescents with a first psychotic episode. J Am Acad Child Adolesc Psychiatry, 2005, 44(11): 1151-1157.

[21] Gogtay N, Sporn A, Clasen LS, et al. Structural brain MRI abnormalities in healthy siblings of patients with childhood-onset schizophrenia. Am J Psychiatry, 2003, 160(3): 569-571.

[22] Sowell ER, Levitt J, Thompson PM, et al. Brain abnormalities in early-onset schizophrenia spectrum disorder observed with statistical parametric mapping of structural magnetic resonance images. Am J Psychiatry, 2000, 157(9): 1475-1484.

[23] ZHU Junjing, GUO Suqin, LIANG Yinghui, et al. Association of abnormal behavior with brain structure in first-episode childhood schizophrenia. Chinese Journal of School Health, 2018, 39(7).

[24] Seitz J, Rathi Y, Lyall A, et al. Alteration of gray matter microstructure in schizophrenia. Brain Imaging Behav, 2018, 12(1): 54-63.

[25] Quinn M, McHugo M, Armstrong K, et al. Impact of substance use disorder on gray matter volume in schizophrenia. Psychiatry Res Neuroimaging, 2018, 280: 9-14.

[26] Sun T, Zhao P, Jiang X, et al. Distinct Associations of Cognitive Impairments and Reduced Gray Matter Volumes in Remitted Patients with Schizophrenia and Bipolar Disorder. Neural Plast, 2020, 2020: 8859388.

[27] Wang X, Zhao N, Shi J, et al. Discussion on the Application of Multi-modal Magnetic Resonance Imaging Fusion in Schizophrenia. J Med Syst, 2019, 43(5): 131.

[28] Schwarz E, Doan NT, Pergola G, et al. Reproducible grey matter patterns index a multivariate, global alteration of brain structure in schizophrenia and bipolar disorder. Transl Psychiatry, 2019, 9(1): 12.

[29] Núñez C, Stephan-Otto C, Usall J, et al. Neutrophil Count Is Associated With Reduced Gray Matter and Enlarged Ventricles in First-Episode Psychosis. Schizophr Bull, 2019, 45(4): 846-858.

[30] Lin Y, Li M, Zhou Y, et al. Age-Related Reduction in Cortical Thickness in First-Episode Treatment-Naïve Patients with Schizophrenia. Neurosci Bull, 2019, 35(4): 688-696.

[31] Lei W, Kirkpatrick B, Wang Q, et al. Progressive brain structural changes after the first year of treatment in first-episode treatment-naive patients with deficit or nondeficit schizophrenia. Psychiatry Res Neuroimaging, 2019, 288: 12-20.

[32] Lee KH, Oh H, Suh JS, et al. Functional and Structural Connectivity of the Cerebellar Nuclei With the Striatum and Cerebral Cortex in First-Episode Psychosis. J Neuropsychiatry Clin Neurosci, 2019, 31(2): 143-151.

[33] Fan F, Xiang H, Tan S, et al. Subcortical structures and cognitive dysfunction in first episode schizophrenia. Psychiatry Res Neuroimaging, 2019, 286: 69-75.

[34] Escarti MJ, Garcia-Marti G, Sanz-Requena R, et al. Auditory hallucinations in first-episode psychosis: A voxel-based morphometry study. Schizophr Res, 2019, 209: 148-155.

[35] Chen J, Calhoun VD, Lin D, et al. Shared Genetic Risk of Schizophrenia and Gray Matter Reduction in 6p22.1. Schizophr Bull, 2019, 45(1): 222-232.

[36] Zhao C, Zhu J, Liu X, et al. Structural and functional brain abnormalities in schizophrenia: A cross-sectional study at different stages of the disease. Prog Neuropsychopharmacol Biol Psychiatry, 2018, 83: 27-32.

[37] Cancel A, Comte M, Truillet R, et al. Childhood neglect predicts disorganization in schizophrenia through grey matter decrease in dorsolateral prefrontal cortex. Acta Psychiatr Scand, 2015, 132(4): 244-256.

[38] Salvador R, Radua J, Canales-Rodríguez EJ, et al. Evaluation of machine learning algorithms and structural features for optimal MRI-based diagnostic prediction in psychosis. PLoS One, 2017, 12(4): e0175683.

[39] Rus OG, Reess TJ, Wagner G, et al. Structural alterations in patients with obsessive-compulsive disorder: a surface-based analysis of cortical volume, surface area and thickness. J Psychiatry Neurosci, 2017, 42(6): 395-403.

[40] Núñez C, Paipa N, Senior C, et al. Global brain asymmetry is increased in schizophrenia and related to avolition. Acta Psychiatr Scand, 2017, 135(5): 448-459.

[41] Watsky RE, Pollard KL, Greenstein D, et al. Severity of Cortical Thinning Correlates With Schizophrenia Spectrum Symptoms. J Am Acad Child Adolesc Psychiatry, 2016, 55(2): 130-136.

[42] Schnack HG, van Haren NE, Nieuwenhuis M, et al. Accelerated Brain Aging in Schizophrenia: A Longitudinal Pattern Recognition Study. Am J Psychiatry, 2016, 173(6): 607-616.

[43] Iwashiro N, Koike S, Satomura Y, et al. Association between impaired brain activity and volume at the sub-region of Broca's area in ultra-high risk and first-episode schizophrenia: A multi-modal neuroimaging study. Schizophr Res, 2016, 172(1-3): 9-15.

[44] Hu X, Liu Q, Li B, et al. Multivariate pattern analysis of obsessive-compulsive disorder using structural neuroanatomy. Eur Neuropsychopharmacol, 2016, 26(2): 246-254.

[45] Vohs JL, Hummer TA, Yung MG, et al. Metacognition in Early Phase Psychosis: Toward Understanding Neural Substrates. Int J Mol Sci, 2015, 16(7): 14640-14654.

[46] Tordesillas-Gutierrez D, Koutsouleris N, Roiz-Santiañez R, et al. Grey matter volume differences in non-affective psychosis and the effects of age of onset on grey matter volumes: A voxelwise study. Schizophr Res, 2015, 164(1-3): 74-82.

[47] Tijms BM, Sprooten E, Job D, et al. Grey matter networks in people at increased familial risk for schizophrenia. Schizophr Res, 2015, 168(1-2): 1-8.

[48] Tao H, Wong GH, Zhang H, et al. Grey matter morphological anomalies in the caudate head in first-episode psychosis patients with delusions of reference. Psychiatry Res, 2015, 233(1): 57-63.

[49] Huang P, Xi Y, Lu ZL, et al. Decreased bilateral thalamic gray matter volume in first-episode schizophrenia with prominent hallucinatory symptoms: A volumetric MRI study. Sci Rep, 2015, 5: 14505.

[50] Guo W, Liu F, Xiao C, et al. Dissociation of anatomical and functional alterations of the default-mode network in first-episode, drug-naive schizophrenia. Clin Neurophysiol, 2015, 126(12): 2276-2281.

[51] Guo W, Liu F, Liu J, et al. Abnormal causal connectivity by structural deficits in first-episode, drug-naive schizophrenia at rest. Schizophr Bull, 2015, 41(1): 57-65.

[52] Gong Q, Dazzan P, Scarpazza C, et al. A Neuroanatomical Signature for Schizophrenia Across Different Ethnic Groups. Schizophr Bull, 2015, 41(6): 1266-1275.

[53] Ferro A, Roiz-Santiáñez R, Ortíz-García de la Foz V, et al. A cross-sectional and longitudinal structural magnetic resonance imaging study of the post-central gyrus in first-episode schizophrenia patients. Psychiatry Res, 2015, 231(1): 42-49.

[54] Ahmed M, Cannon DM, Scanlon C, et al. Progressive Brain Atrophy and Cortical Thinning in Schizophrenia after Commencing Clozapine Treatment. Neuropsychopharmacology, 2015, 40(10): 2409-2417.

[55] Radeloff D, Ciaramidaro A, Siniatchkin M, et al. Structural alterations of the social brain: a comparison between schizophrenia and autism. PLoS One, 2014, 9(9): e106539.

[56] Gong X, Lu W, Kendrick KM, et al. A brain-wide association study of DISC1 genetic variants reveals a relationship with the structure and functional connectivity of the precuneus in schizophrenia. Hum Brain Mapp, 2014, 35(11): 5414-5430.

[57] Voineskos AN, Foussias G, Lerch J, et al. Neuroimaging evidence for the deficit subtype of schizophrenia. JAMA Psychiatry, 2013, 70(5): 472-480.

[58] Tang W, Li B, Huang X, et al. Morphometric brain characterization of refractory obsessive-compulsive disorder: diffeomorphic anatomic registration using exponentiated Lie algebra. Prog Neuropsychopharmacol Biol Psychiatry, 2013, 46: 126-131.

[59] Kumari V, Gudjonsson GH, Raghuvanshi S, et al. Reduced thalamic volume in men with antisocial personality disorder or schizophrenia and a history of serious violence and childhood abuse. Eur Psychiatry, 2013, 28(4): 225-234.

[60] Hou J, Song L, Zhang W, et al. Morphologic and functional connectivity alterations of corticostriatal and default mode network in treatment-naïve patients with obsessive-compulsive disorder. PLoS One, 2013, 8(12): e83931.

[61] Guo X, Li J, Wei Q, et al. Duration of untreated psychosis is associated with temporal and occipitotemporal gray matter volume decrease in treatment naïve schizophrenia. PLoS One, 2013, 8(12): e83679.

[62] Goghari VM, Smith GN, Honer WG, et al. Effects of eight weeks of atypical antipsychotic treatment on middle frontal thickness in drug-naïve first-episode psychosis patients. Schizophr Res, 2013, 149(1-3): 149-155.

[63] Fan Q, Palaniyappan L, Tan L, et al. Surface anatomical profile of the cerebral cortex in obsessive-compulsive disorder: a study of cortical thickness, folding and surface area. Psychol Med, 2013, 43(5): 1081-1091.

[64] Wittfoth M, Bornmann S, Peschel T, et al. Lateral frontal cortex volume reduction in Tourette syndrome revealed by VBM. BMC Neurosci, 2012, 13: 17.

[65] Watson DR, Anderson JM, Bai F, et al. A voxel based morphometry study investigating brain structural changes in first episode psychosis. Behav Brain Res, 2012, 227(1): 91-99.

[66] Roiz-Santiáñez R, Tordesillas-Gutiérrez D, Ortíz-García de la Foz V, et al. Effect of antipsychotic drugs on cortical thickness. A randomized controlled one-year follow-up study of haloperidol, risperidone and olanzapine. Schizophr Res, 2012, 141(1): 22-28.

[67] Li M, Chen Z, Deng W, et al. Volume increases in putamen associated with positive symptom reduction in previously drug-naive schizophrenia after 6 weeks antipsychotic treatment. Psychol Med, 2012, 42(7): 1475-1483.

[68] Dazzan P, Soulsby B, Mechelli A, et al. Volumetric abnormalities predating the onset of schizophrenia and affective psychoses: an MRI study in subjects at ultrahigh risk of psychosis. Schizophr Bull, 2012, 38(5): 1083-1091.

[69] Cobia DJ, Smith MJ, Wang L, et al. Longitudinal progression of frontal and temporal lobe changes in schizophrenia. Schizophr Res, 2012, 139(1-3): 1-6.

[70] Boos HB, Cahn W, van Haren NE, et al. Focal and global brain measurements in siblings of patients with schizophrenia. Schizophr Bull, 2012, 38(4): 814-825.

[71] Takahashi T, Zhou SY, Nakamura K, et al. A follow-up MRI study of the fusiform gyrus and middle and inferior temporal gyri in schizophrenia spectrum. Prog Neuropsychopharmacol Biol Psychiatry, 2011, 35(8): 1957-1964.

[72] Malla AK, Bodnar M, Joober R, et al. Duration of untreated psychosis is associated with orbital-frontal grey matter volume reductions in first episode psychosis. Schizophr Res, 2011, 125(1): 13-20.

[73] Price G, Cercignani M, Chu EM, et al. Brain pathology in first-episode psychosis: magnetization transfer imaging provides additional information to MRI measurements of volume loss. Neuroimage, 2010, 49(1): 185-192.

[74] Horga G, Bernacer J, Dusi N, et al. Correlations between ventricular enlargement and gray and white matter volumes of cortex, thalamus, striatum, and internal capsule in schizophrenia. Eur Arch Psychiatry Clin Neurosci, 2011, 261(7): 467-476.

[75] Moriya J, Kakeda S, Abe O, et al. Gray and white matter volumetric and diffusion tensor imaging (DTI) analyses in the early stage of first-episode schizophrenia. Schizophr Res, 2010, 116(2-3): 196-203.

[76] Harms MP, Wang L, Campanella C, et al. Structural abnormalities in gyri of the prefrontal cortex in individuals with schizophrenia and their unaffected siblings. Br J Psychiatry, 2010, 196(2): 150-157.

[77] Witthaus H, Kaufmann C, Bohner G, et al. Gray matter abnormalities in subjects at ultra-high risk for schizophrenia and first-episode schizophrenic patients compared to healthy controls. Psychiatry Res, 2009, 173(3): 163-169.

[78] Whitford TJ, Farrow TF, Williams LM, et al. Delusions and dorso-medial frontal cortex volume in first-episode schizophrenia: a voxel-based morphometry study. Psychiatry Res, 2009, 172(3): 175-179.

[79] Lui S, Deng W, Huang X, et al. Neuroanatomical differences between familial and sporadic schizophrenia and their parents: an optimized voxel-based morphometry study. Psychiatry Res, 2009, 171(2): 71-81.

[80] Herold R, Feldmann A, Simon M, et al. Regional gray matter reduction and theory of mind deficit in the early phase of schizophrenia: a voxel-based morphometric study. Acta Psychiatr Scand, 2009, 119(3): 199-208.

[81] Crespo-Facorro B, Roiz-Santiáñez R, Pérez-Iglesias R, et al. Specific brain structural abnormalities in first-episode schizophrenia. A comparative study with patients with schizophreniform disorder, non-schizophrenic non-affective psychoses and healthy volunteers. Schizophr Res, 2009, 115(2-3): 191-201.

[82] Zipparo L, Whitford TJ, Redoblado Hodge MA, et al. Investigating the neuropsychological and neuroanatomical changes that occur over the first 2-3 years of illness in patients with first-episode schizophrenia. Prog Neuropsychopharmacol Biol Psychiatry, 2008, 32(2): 531-538.

[83] Meisenzahl EM, Koutsouleris N, Bottlender R, et al. Structural brain alterations at different stages of schizophrenia: a voxel-based morphometric study. Schizophr Res, 2008, 104(1-3): 44-60.

[84] Bangalore SS, Prasad KM, Montrose DM, et al. Cannabis use and brain structural alterations in first episode schizophrenia--a region of interest, voxel based morphometric study. Schizophr Res, 2008, 99(1-3): 1-6.

[85] Whitford TJ, Farrow TF, Rennie CJ, et al. Longitudinal changes in neuroanatomy and neural activity in early schizophrenia. Neuroreport, 2007, 18(5): 435-439.

[86] Schaufelberger MS, Duran FL, Lappin JM, et al. Grey matter abnormalities in Brazilians with first-episode psychosis. Br J Psychiatry Suppl, 2007, 51: s117-122.

[87] Whitford TJ, Grieve SM, Farrow TF, et al. Progressive grey matter atrophy over the first 2-3 years of illness in first-episode schizophrenia: a tensor-based morphometry study. Neuroimage, 2006, 32(2): 511-519.

[88] Antonova E, Kumari V, Morris R, et al. The relationship of structural alterations to cognitive deficits in schizophrenia: a voxel-based morphometry study. Biol Psychiatry, 2005, 58(6): 457-467.

[89] Kawasaki Y, Suzuki M, Nohara S, et al. Structural brain differences in patients with schizophrenia and schizotypal disorder demonstrated by voxel-based morphometry. Eur Arch Psychiatry Clin Neurosci, 2004, 254(6): 406-414.

[90] Suzuki M, Nohara S, Hagino H, et al. Regional changes in brain gray and white matter in patients with schizophrenia demonstrated with voxel-based analysis of MRI. Schizophr Res, 2002, 55(1-2): 41-54.

[91] Chang M, Womer FY, Bai C, et al. Voxel-Based Morphometry in Individuals at Genetic High Risk for Schizophrenia and Patients with Schizophrenia during Their First Episode of Psychosis. PLoS One, 2016, 11(10): e0163749.

[92] WEI Qin-ling, KANG Zhuang, WU Xiao-li, et al. Changes of Gray Matter Volumes in Patients with Early-onset Schizophrenia. Journal of Sun Yat-sen University(Medical Sciences), 2011, 32(4).

[93] Abush H, Ghose S, Van Enkevort EA, et al. Associations between adolescent cannabis use and brain structure in psychosis. Psychiatry Res Neuroimaging, 2018, 276: 53-64.

[94] Zarogianni E, Storkey AJ, Johnstone EC, et al. Improved individualized prediction of schizophrenia in subjects at familial high risk, based on neuroanatomical data, schizotypal and neurocognitive features. Schizophr Res, 2017, 181: 6-12.

[95] Sugranyes G, de la Serna E, Ilzarbe D, et al. Brain structural trajectories in youth at familial risk for schizophrenia or bipolar disorder according to development of psychosis spectrum symptoms. J Child Psychol Psychiatry, 2021, 62(6): 780-789.

[96] Shan XX, Ou YP, Pan P, et al. Increased frontal gray matter volume in individuals with prodromal psychosis. CNS Neurosci Ther, 2019, 25(9): 987-994.

[97] Kong L, Cui H, Zhang T, et al. Neurological soft signs and grey matter abnormalities in individuals with ultra-high risk for psychosis. Psych J, 2019, 8(2): 252-260.

[98] de Zwarte SMC, Brouwer RM, Tsouli A, et al. Running in the Family? Structural Brain Abnormalities and IQ in Offspring, Siblings, Parents, and Co-twins of Patients with Schizophrenia. Schizophr Bull, 2019, 45(6): 1209-1217.

[99] Evans DW, Michael AM, Ularević M, et al. Neural substrates of a schizotypal spectrum in typically-developing children: Further evidence of a normal-pathological continuum. Behav Brain Res, 2016, 315: 141-146.

[100] Riddle K, Cascio CJ, Woodward ND. Brain structure in autism: a voxel-based morphometry analysis of the Autism Brain Imaging Database Exchange (ABIDE). Brain Imaging Behav, 2017, 11(2): 541-551.

[101] Dukart J, Smieskova R, Harrisberger F, et al. Age-related brain structural alterations as an intermediate phenotype of psychosis. J Psychiatry Neurosci, 2017, 42(5): 307-319.

[102] Satterthwaite TD, Wolf DH, Calkins ME, et al. Structural Brain Abnormalities in Youth With Psychosis Spectrum Symptoms. JAMA Psychiatry, 2016, 73(5): 515-524.

[103] McKechanie AG, Moorhead TW, Stanfield AC, et al. Negative symptoms and longitudinal grey matter tissue loss in adolescents at risk of psychosis: preliminary findings from a 6-year follow-up study. Br J Psychiatry, 2016, 208(6): 565-570.

[104] Brent BK, Rosso IM, Thermenos HW, et al. Alterations of lateral temporal cortical gray matter and facial memory as vulnerability indicators for schizophrenia: An MRI study in youth at familial high-risk for schizophrenia. Schizophr Res, 2016, 170(1): 123-129.

[105] Wagshal D, Knowlton BJ, Cohen JR, et al. Cognitive correlates of gray matter abnormalities in adolescent siblings of patients with childhood-onset schizophrenia. Schizophr Res, 2015, 161(2-3): 345-350.

[106] Klauser P, Zhou J, Lim JK, et al. Lack of Evidence for Regional Brain Volume or Cortical Thickness Abnormalities in Youths at Clinical High Risk for Psychosis: Findings From the Longitudinal Youth at Risk Study. Schizophr Bull, 2015, 41(6): 1285-1293.

[107] Guo W, Song Y, Liu F, et al. Dissociation of functional and anatomical brain abnormalities in unaffected siblings of schizophrenia patients. Clin Neurophysiol, 2015, 126(5): 927-932.

[108] Moorhead TW, Stanfield AC, McKechanie AG, et al. Longitudinal gray matter change in young people who are at enhanced risk of schizophrenia due to intellectual impairment. Biol Psychiatry, 2013, 73(10): 985-992.

[109] Lincoln SH, Hooker CI. Neural structure and social dysfunction in individuals at clinical high risk for psychosis. Psychiatry Res, 2014, 224(3): 152-158.

[110] Montigny C, Castellanos-Ryan N, Whelan R, et al. A phenotypic structure and neural correlates of compulsive behaviors in adolescents. PLoS One, 2013, 8(11): e80151.

[111] Cullen AE, De Brito SA, Gregory SL, et al. Temporal lobe volume abnormalities precede the prodrome: a study of children presenting antecedents of schizophrenia. Schizophr Bull, 2013, 39(6): 1318-1327.

[112] Bhojraj TS, Francis AN, Montrose DM, et al. Grey matter and cognitive deficits in young relatives of schizophrenia patients. Neuroimage, 2011, 54 Suppl 1(0 1): S287-292.

[113] Jacobson S, Kelleher I, Harley M, et al. Structural and functional brain correlates of subclinical psychotic symptoms in 11-13 year old schoolchildren. Neuroimage, 2010, 49(2): 1875-1885.

[114] Moorhead TW, Stanfield A, Spencer M, et al. Progressive temporal lobe grey matter loss in adolescents with schizotypal traits and mild intellectual impairment. Psychiatry Res, 2009, 174(2): 105-109.

[115] Chen J, Silk T, Seal M, et al. Widespread decreased grey and white matter in paediatric obsessive-compulsive disorder (OCD): a voxel-based morphometric MRI study. Psychiatry Res, 2013, 213(1): 11-17.

[116] Burke L, Androutsos C, Jogia J, et al. The Maudsley Early Onset Schizophrenia Study: the effect of age of onset and illness duration on fronto-parietal gray matter. Eur Psychiatry, 2008, 23(4): 233-236.

[117] LI Hui. Effect of Ziprasidone on Brain Neurological Function and Structure in First-episode Adolescent Schizophrenic Patients. Chinese Journal of Modern Applied Pharmacy, 2020, 37(07).

[118] Douaud G, Mackay C, Andersson J, et al. Schizophrenia delays and alters maturation of the brain in adolescence. Brain, 2009, 132(Pt 9): 2437-2448.

[119] Voets NL, Hough MG, Douaud G, et al. Evidence for abnormalities of cortical development in adolescent-onset schizophrenia. Neuroimage, 2008, 43(4): 665-675.

[120] Zhang Lin, Wang Xiao, Zhang Yan, et al. Cerebral grey matter changes of pre- and post-treatment in first-episode drug-naive adolescents schizophrenia. National Medical Journal of China, 2018, 98(47).

[121] ZHANG Chengcheng, LI Mingli, LI Na, et al. Abnormal grey matter changes in first-episode antipsychotic-naïve patients with adolescent-onset schizophrenia. Chinese Journal of Nervous and Mental Diseases, 2014, 40(6).

[122] WANG Xiao, ZHANG Yan, SUN Meng-xi, et al. The correlations between brain gray matter volume and cognitive function in first-episode adolescents with schizophrenia. Journal of Clinical Psychiatry, 2016, 12(6).
